# Correction: Understanding COVID-19 vaccine hesitancy in Meghalaya, India: Multiple correspondence and agglomerative hierarchical cluster analyses

**DOI:** 10.1371/journal.pgph.0004261

**Published:** 2025-01-29

**Authors:** The PLOS ONE Staff

There are errors in the Author Contributions. The correct contributions are:

**Conceptualization:** Sooyoung Kim, Rajiv Sarkar, Sampath Kumar, Yesim Tozan, Sandra Albert

**Data curation:** Rajiv Sarkar, Melissa Glenda Lewis, Sandra Albert

**Formal analysis:** Sooyoung Kim

**Investigation:** Sooyoung Kim, Rajiv Sarkar

**Methodology:** Sooyoung Kim, Rajiv Sarkar, Melissa Glenda Lewis, Yesim Tozan

**Project administration:** Yesim Tozan, Sandra Albert

**Supervision:** Yesim Tozan, Sandra Albert

**Validation:** Sooyoung Kim, Rajiv Sarkar

**Visualization:** Sooyoung Kim

**Writing–original draft:** Sooyoung Kim

**Writing–review & editing:** Sooyoung Kim, Rajiv Sarkar, Sampath Kumar, Melissa Glenda Lewis, Yesim Tozan, Sandra Albert

The publisher apologizes for the error.
