# Supplementary material for: Understanding COVID-19 vaccine hesitancy in Meghalaya, India: Multiple correspondence and agglomerative hierarchical cluster analyses
Source: PLOS Glob Public Health. 2024 Feb 27;4(2):e0002250. doi: 10.1371/journal.pgph.0002250 (PMC10898751; doi:10.1371/journal.pgph.0002250)
Supplement: S1 Text — Table A. Vaccine hesitancy questionnaire Table B. Eigen value and percentage of variance explained by each dimension in the multiple correspondence analysis Fig A. Variable coordinates of multiple correspondence analysis (Dimensions 1 and 3) Fig B. Variable coordinates of multiple correspondence analysis (Dimensions 1 and 4) Fig C. Variable coordinates of multiple correspondence analysis (Dimensions 1 and 5) Fig D. Variable coordinates of multiple correspondence analysis (Dimensions 2 and 3) Fig E. Variable coordinates of multiple correspondence analysis (Dimensions 2 and 4) Fig F. Variable coordinates of multiple correspondence analysis (Dimensions 2 and 5) Fig G. Variable coordinates of multiple correspondence analysis (Dimensions 3 and 4) Fig H. Variable coordinates of multiple correspondence analysis (Dimensions 3 and 5) Fig I. Variable coordinates of multiple correspondence analysis (Dimensions 4 and 5) Table C. Demographic and socioeconomic characteristics of survey respondents in each cluster (DOCX) [file pgph.0002250.s002.docx]

Table A. COVID-19 vaccine hesitancy questionnaire

| Question | Response |
| --- | --- |
| The vaccination centre is too far away | (Yes/No) |
| I do not like going to the hospitals | (Yes/No) |
| I find the COVID 19 vaccination registration process difficult | (Yes/No) |
| I am worried about the cost of the vaccine | (Yes/No) |
| I want to decide which vaccine to take | (Yes/No) |
| I am confused by different messages from different sources | (Yes/No) |
| I currently do not have trustworthy sources of information on covid vaccines | (Yes/No) |
| My family members advising against taking the vaccination | (Yes/No) |
| I have seen messages on whatsapp and others that cause concern | (Yes/No) |
| Influential leaders that I listen to do not support the vaccine | (Yes/No) |
| None of my friends/relatives have taken it | (Yes/No) |
| I am worried about the side effects after vaccination | (Yes/No) |
| I have a fear of pain or needles | (Yes/No) |
| I think that the COVID-19 vaccines are not safe | (Yes/No) |
| I think that vaccine may cause infertility and impotence | (Yes/No) |
| I think that the COVID-19 vaccine might affect future pregnancy | (Yes/No) |
| I believe that natural immunity is sufficient | (Yes/No) |
| I think I have developed immunity against the virus | (Yes/No) |

Table B. Eigen value and percentage of variance explained by each dimension in the multiple correspondence analysis

| Dimension | eigenvalue | percentage of variance | cumulative percentage of variance |
| --- | --- | --- | --- |
| Dim.1 | 0.203 | 9.198 | 9.198 |
| Dim.2 | 0.130 | 5.887 | 15.085 |
| Dim.3 | 0.104 | 4.713 | 19.798 |
| Dim.4 | 0.081 | 3.668 | 23.465 |
| Dim.5 | 0.075 | 3.373 | 26.838 |
| Dim.6 | 0.072 | 3.250 | 30.089 |
| Dim.7 | 0.066 | 2.991 | 33.080 |
| Dim.8 | 0.061 | 2.766 | 35.846 |
| Dim.9 | 0.058 | 2.625 | 38.471 |
| Dim.10 | 0.051 | 2.293 | 40.764 |
| Dim.11 | 0.049 | 2.219 | 42.983 |
| Dim.12 | 0.048 | 2.174 | 45.157 |
| Dim.13 | 0.046 | 2.069 | 47.227 |
| Dim.14 | 0.045 | 2.017 | 49.244 |
| Dim.15 | 0.043 | 1.965 | 51.209 |


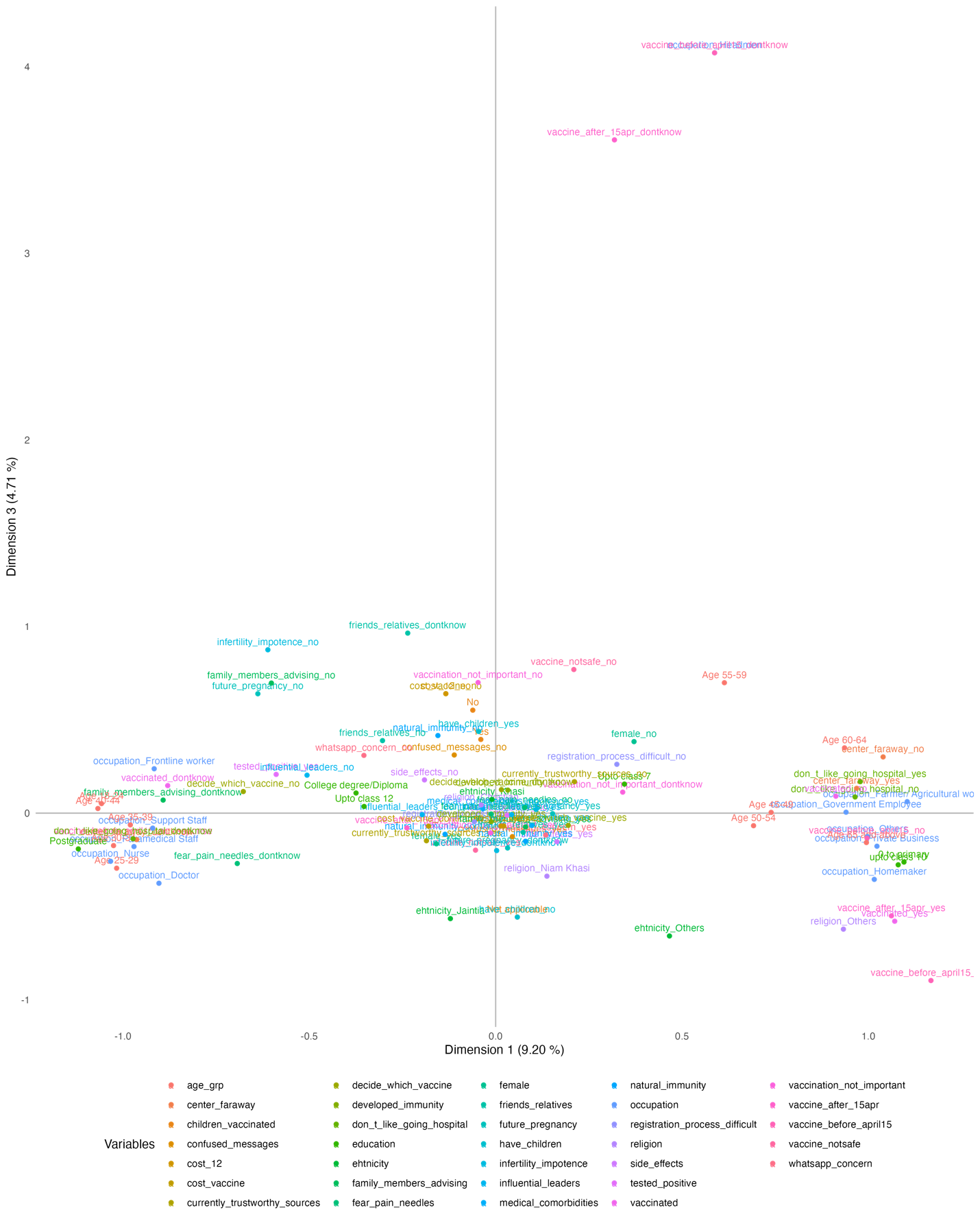


Fig A. Variable coordinates of multiple correspondence analysis (Dimensions 1 and 3)


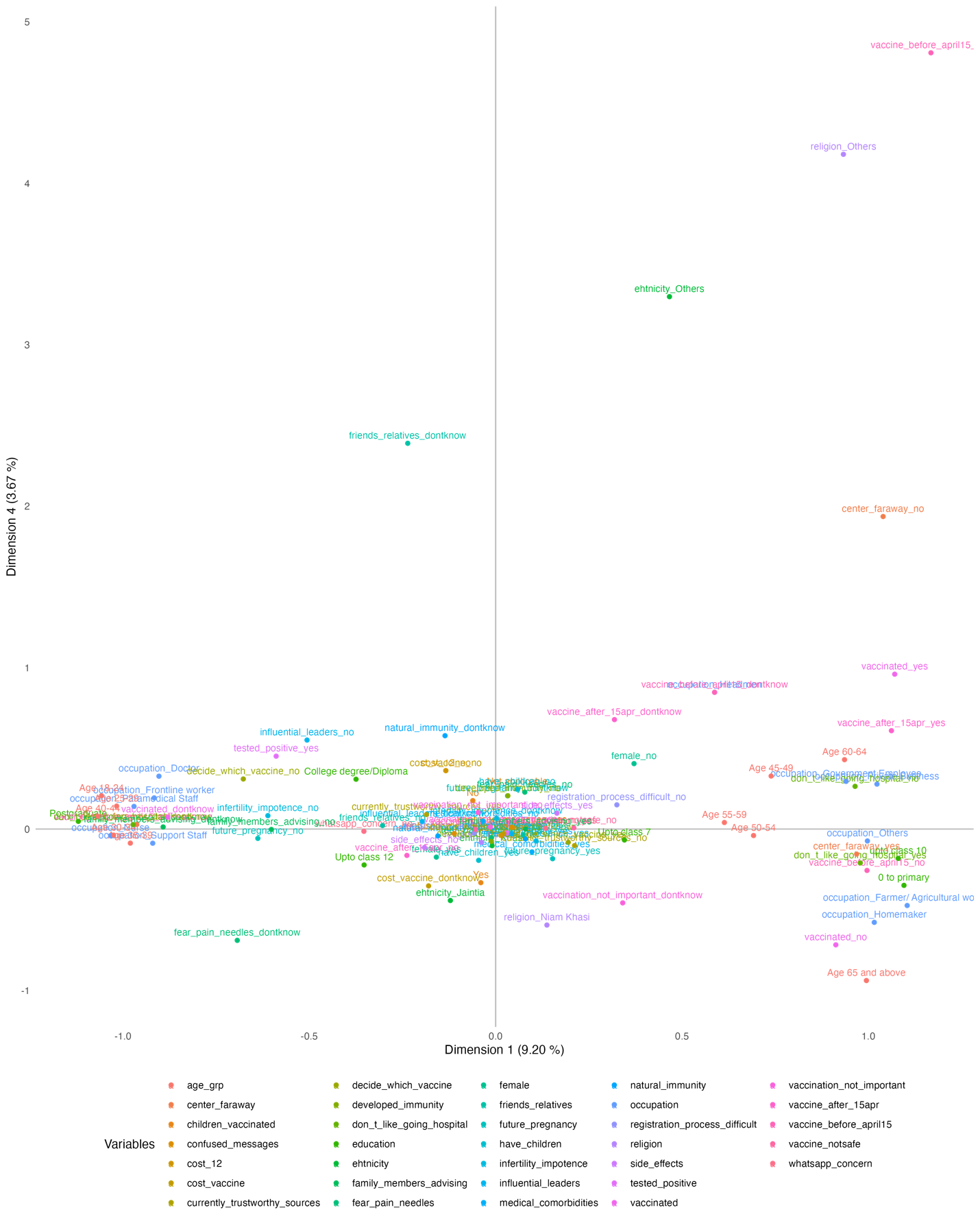


Fig B. Variable coordinates of multiple correspondence analysis (Dimensions 1 and 4)


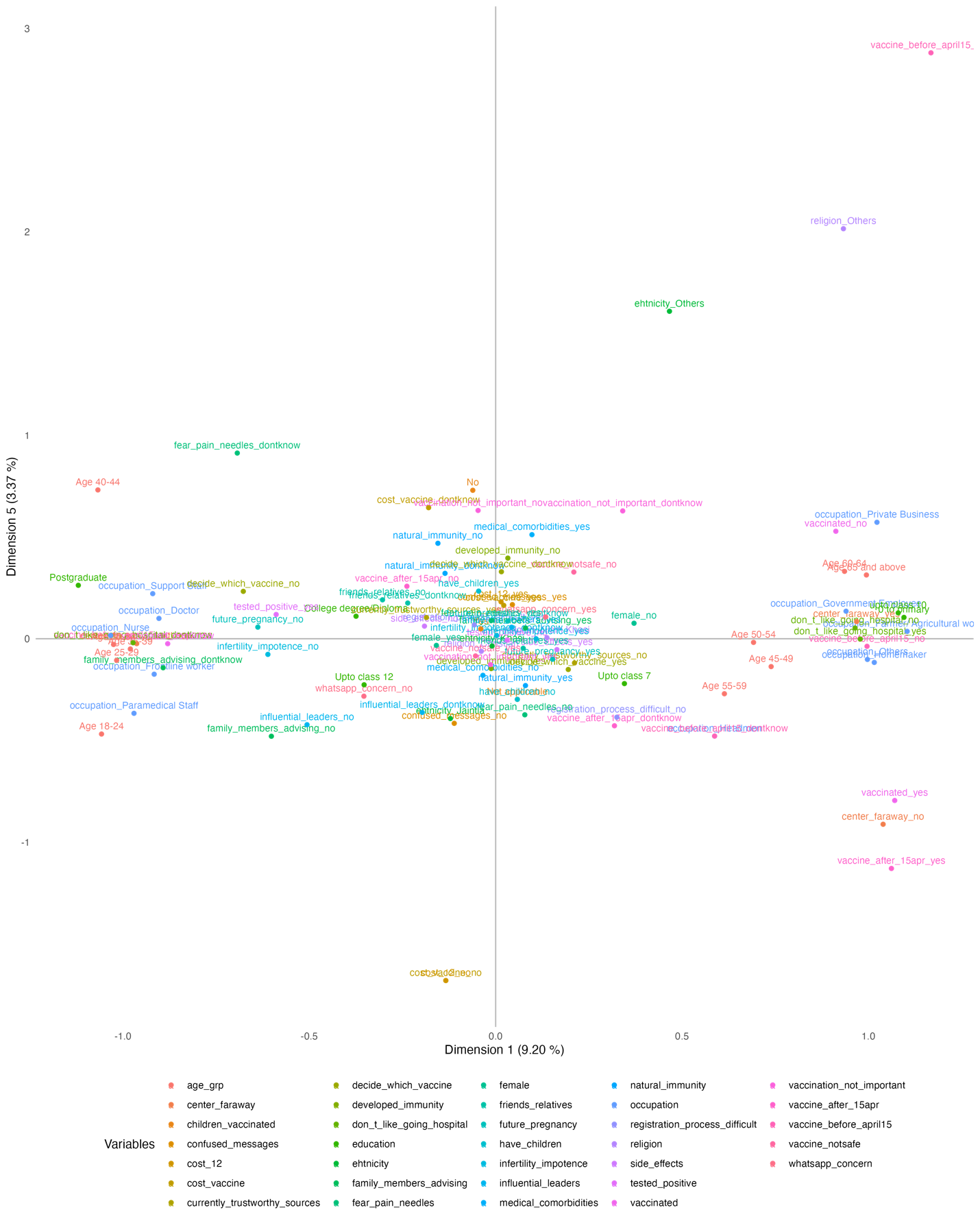


Fig C. Variable coordinates of multiple correspondence analysis (Dimensions 1 and 5)


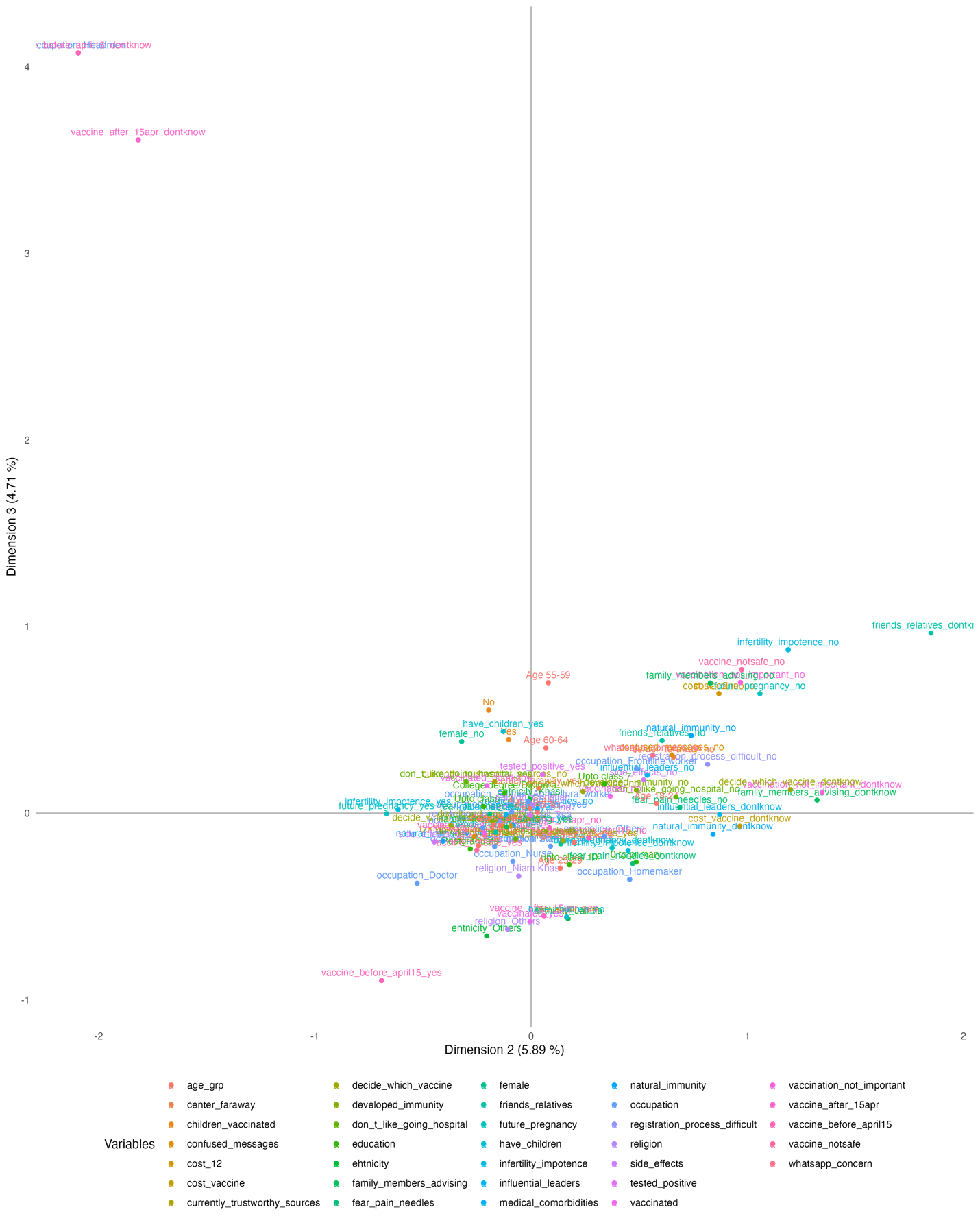


Fig D. Variable coordinates of multiple correspondence analysis (Dimensions 2 and 3)


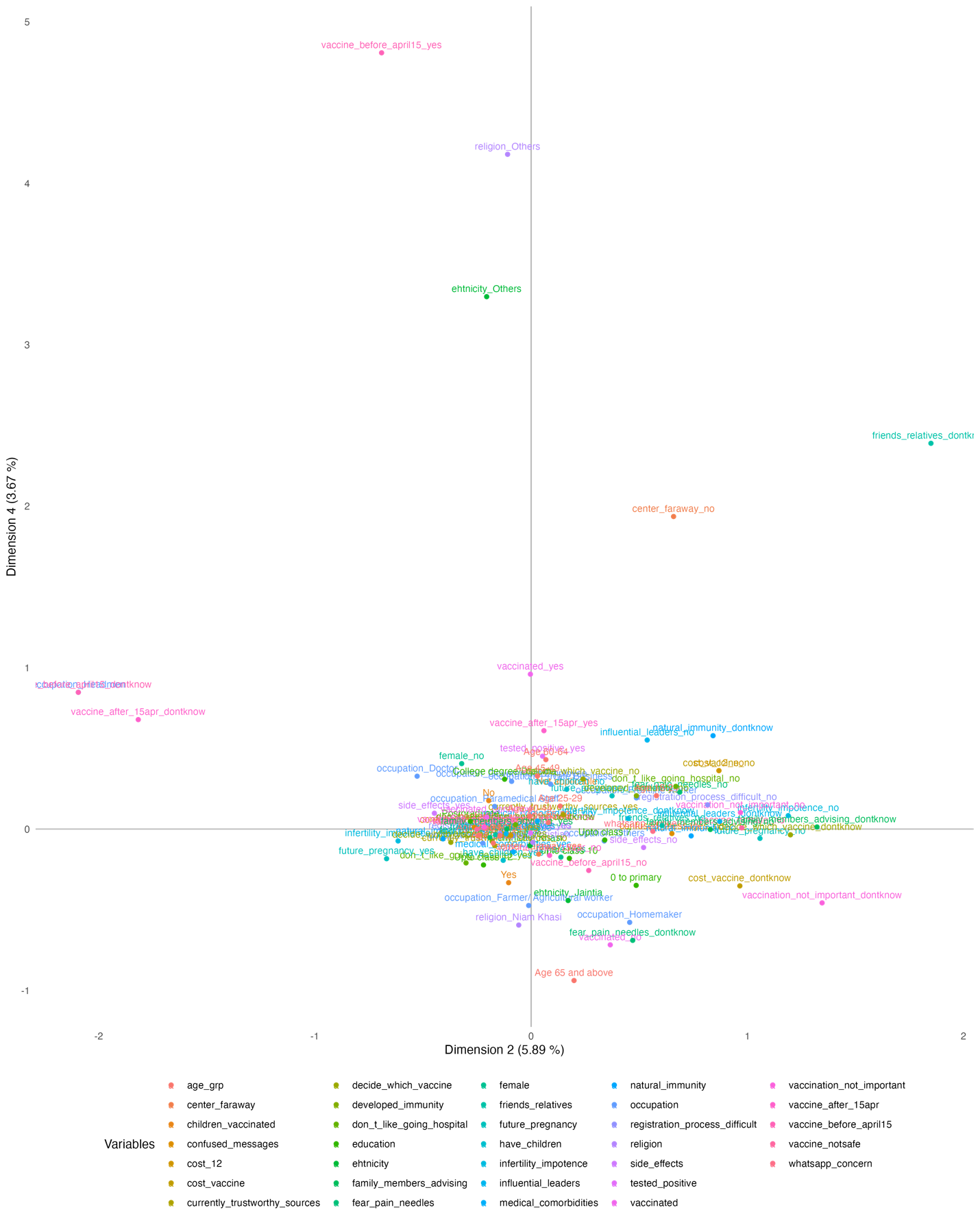


Fig E. Variable coordinates of multiple correspondence analysis (Dimensions 2 and 4)


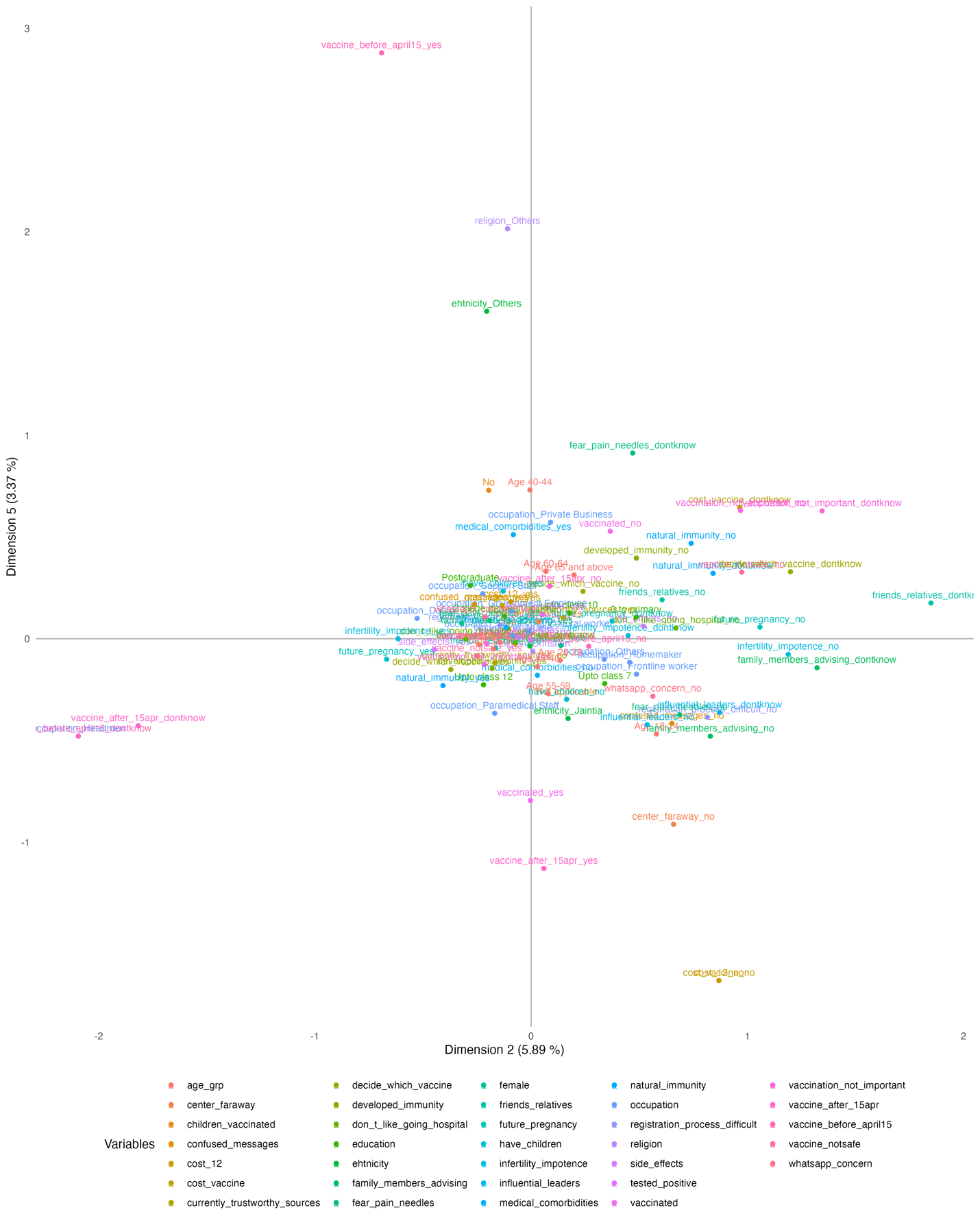


Fig F. Variable coordinates of multiple correspondence analysis (Dimensions 2 and 5)


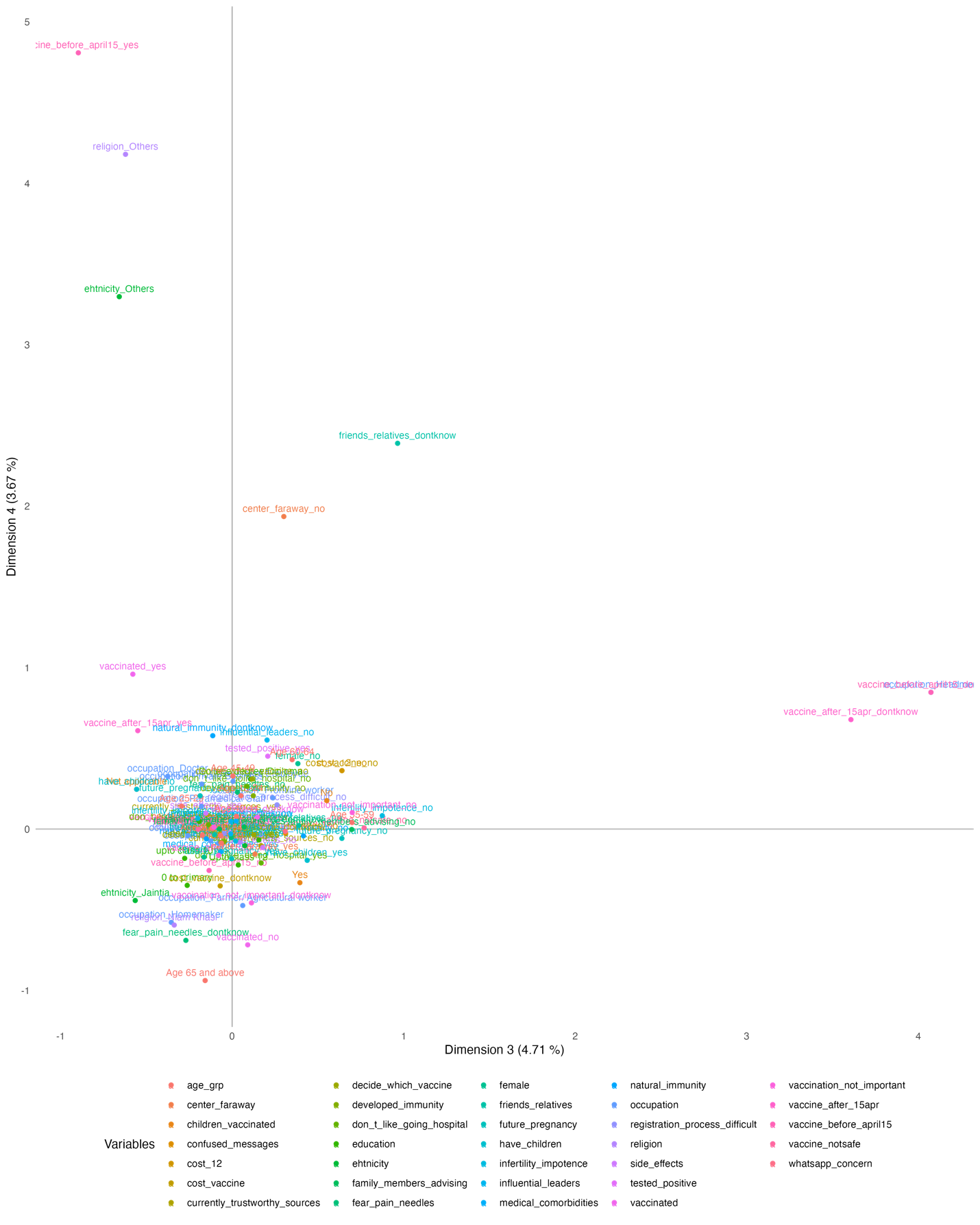


Fig G. Variable coordinates of multiple correspondence analysis (Dimensions 3 and 4)


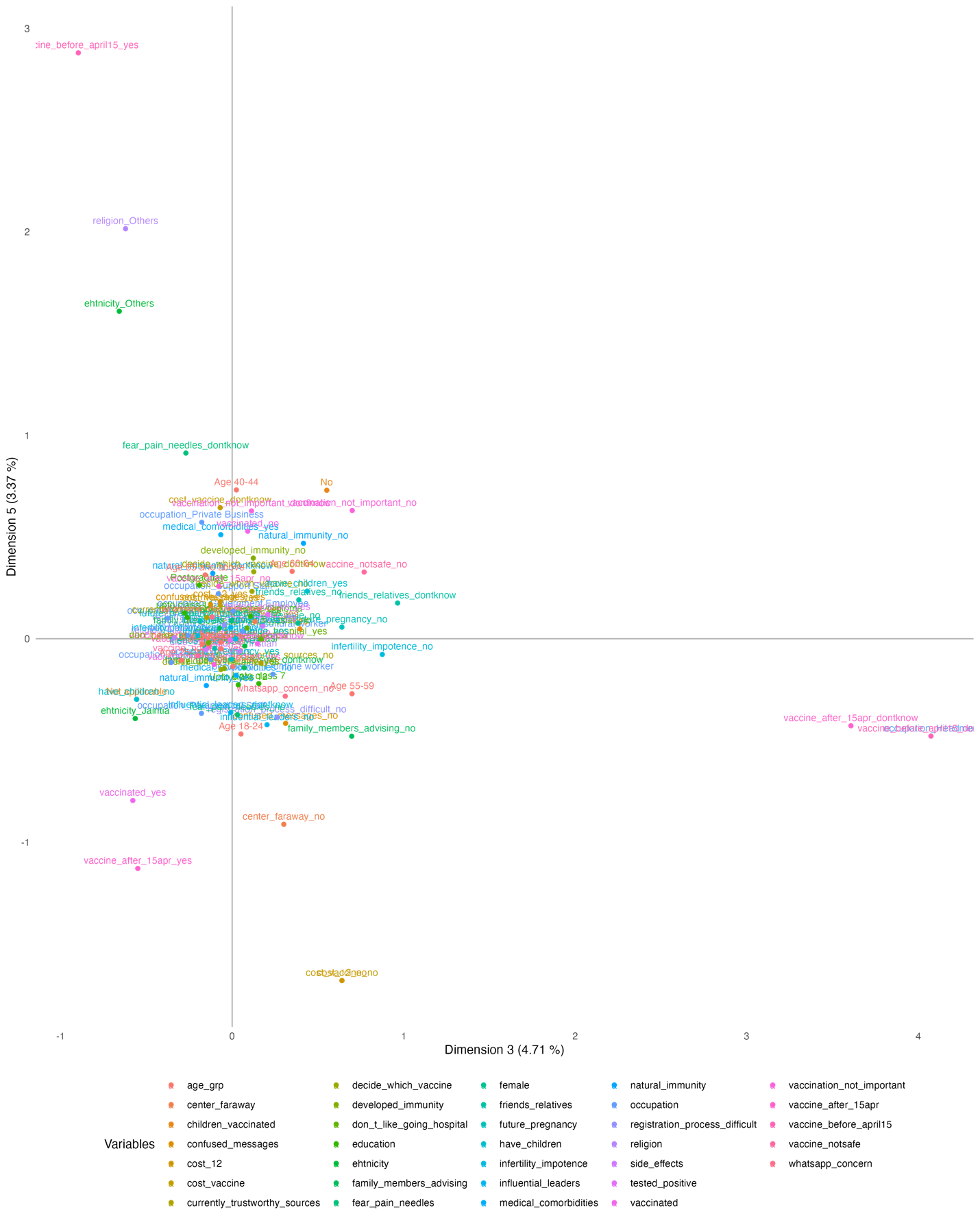


Fig H. Variable coordinates of multiple correspondence analysis (Dimensions 3 and 5)


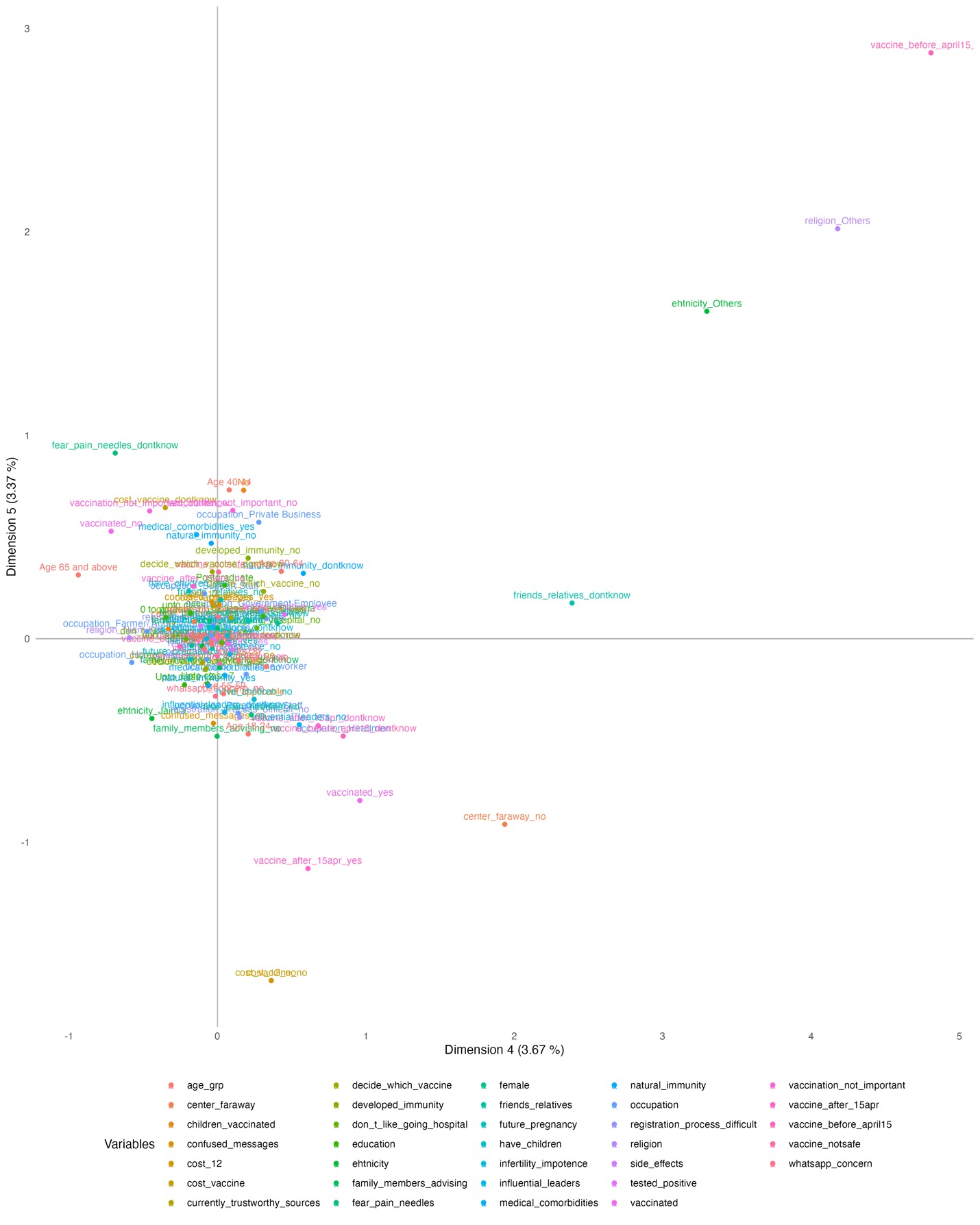


Fig I. Variable coordinates of multiple correspondence analysis (Dimensions 4 and 5)

Table C. Demographic and socioeconomic characteristics of survey respondents in each cluster

|  | **Cluster 1 (N=71)** | **Cluster 2 (N=128)** | **Cluster 3 (N=14)** | **Cluster 4 (N=47)** | **Cluster 5 (N=76)** | **Cluster 6 (N=56)** | **Cluster 7 (N=8)** | **Overall (N=400)** |
| --- | --- | --- | --- | --- | --- | --- | --- | --- |
| **Age group** |  |  |  |  |  |  |  |  |
| 18-24 years | 13 (18.3%) | 7 (5.5%) | 0 (0%) | 0 (0%) | 0 (0%) | 0 (0%) | 0 (0%) | 20 (5.0%) |
| 25-29 years | 17 (23.9%) | 21 (16.4%) | 0 (0%) | 0 (0%) | 0 (0%) | 0 (0%) | 0 (0%) | 38 (9.5%) |
| 30-34 years | 16 (22.5%) | 44 (34.4%) | 0 (0%) | 0 (0%) | 0 (0%) | 0 (0%) | 0 (0%) | 60 (15.0%) |
| 35-39 years | 14 (19.7%) | 27 (21.1%) | 0 (0%) | 0 (0%) | 0 (0%) | 1 (1.8%) | 0 (0%) | 42 (10.5%) |
| 40-44 years | 6 (8.5%) | 8 (6.3%) | 0 (0%) | 0 (0%) | 0 (0%) | 0 (0%) | 0 (0%) | 14 (3.5%) |
| 45-49 years | 3 (4.2%) | 5 (3.9%) | 3 (21.4%) | 11 (23.4%) | 15 (19.7%) | 19 (33.9%) | 2 (25.0%) | 58 (14.5%) |
| 50-54 years | 1 (1.4%) | 10 (7.8%) | 3 (21.4%) | 11 (23.4%) | 18 (23.7%) | 17 (30.4%) | 3 (37.5%) | 63 (15.8%) |
| 55-59 years | 0 (0%) | 5 (3.9%) | 4 (28.6%) | 8 (17.0%) | 6 (7.9%) | 6 (10.7%) | 1 (12.5%) | 30 (7.5%) |
| 60-64 years | 1 (1.4%) | 1 (0.8%) | 4 (28.6%) | 8 (17.0%) | 10 (13.2%) | 9 (16.1%) | 2 (25.0%) | 35 (8.8%) |
| 65 years and above | 0 (0%) | 0 (0%) | 0 (0%) | 9 (19.1%) | 27 (35.5%) | 4 (7.1%) | 0 (0%) | 40 (10.0%) |
| **Sex** |  |  |  |  |  |  |  |  |
| Male | 11 (15.5%) | 29 (22.7%) | 14 (100%) | 17 (36.2%) | 24 (31.6%) | 18 (32.1%) | 7 (87.5%) | 120 (30.0%) |
| Female | 60 (84.5%) | 99 (77.3%) | 0 (0%) | 30 (63.8%) | 52 (68.4%) | 38 (67.9%) | 1 (12.5%) | 280 (70.0%) |
| **Ehtnicity** |  |  |  |  |  |  |  |  |
| Jaintia | 9 (12.7%) | 7 (5.5%) | 0 (0%) | 0 (0%) | 8 (10.5%) | 5 (8.9%) | 0 (0%) | 29 (7.3%) |
| Khasi | 60 (84.5%) | 118 (92.2%) | 14 (100%) | 46 (97.9%) | 68 (89.5%) | 50 (89.3%) | 0 (0%) | 356 (89.0%) |
| Others | 2 (2.8%) | 3 (2.3%) | 0 (0%) | 1 (2.1%) | 0 (0%) | 1 (1.8%) | 8 (100%) | 15 (3.8%) |
| **Religion** |  |  |  |  |  |  |  |  |
| Christian | 68 (95.8%) | 118 (92.2%) | 14 (100%) | 43 (91.5%) | 67 (88.2%) | 52 (92.9%) | 0 (0%) | 362 (90.5%) |
| Niam Khasi | 3 (4.2%) | 9 (7.0%) | 0 (0%) | 3 (6.4%) | 9 (11.8%) | 3 (5.4%) | 0 (0%) | 27 (6.8%) |
| Others | 0 (0%) | 1 (0.8%) | 0 (0%) | 1 (2.1%) | 0 (0%) | 1 (1.8%) | 8 (100%) | 11 (2.8%) |
| **Occupation** |  |  |  |  |  |  |  |  |
| Farmer/ Agricultural worker | 0 (0%) | 0 (0%) | 0 (0%) | 3 (6.4%) | 4 (5.3%) | 2 (3.6%) | 0 (0%) | 9 (2.3%) |
| Government Employee | 0 (0%) | 0 (0%) | 0 (0%) | 9 (19.1%) | 10 (13.2%) | 10 (17.9%) | 2 (25.0%) | 31 (7.8%) |
| Headmen | 0 (0%) | 0 (0%) | 14 (100%) | 0 (0%) | 0 (0%) | 0 (0%) | 0 (0%) | 14 (3.5%) |
| Homemaker | 0 (0%) | 0 (0%) | 0 (0%) | 9 (19.1%) | 21 (27.6%) | 15 (26.8%) | 0 (0%) | 45 (11.3%) |
| Others | 0 (0%) | 0 (0%) | 0 (0%) | 19 (40.4%) | 27 (35.5%) | 20 (35.7%) | 3 (37.5%) | 69 (17.3%) |
| Private Business | 0 (0%) | 0 (0%) | 0 (0%) | 7 (14.9%) | 14 (18.4%) | 8 (14.3%) | 3 (37.5%) | 32 (8.0%) |
| Doctor | 2 (2.8%) | 7 (5.5%) | 0 (0%) | 0 (0%) | 0 (0%) | 0 (0%) | 0 (0%) | 9 (2.3%) |
| Frontline worker | 17 (23.9%) | 10 (7.8%) | 0 (0%) | 0 (0%) | 0 (0%) | 1 (1.8%) | 0 (0%) | 28 (7.0%) |
| Nurse | 25 (35.2%) | 56 (43.8%) | 0 (0%) | 0 (0%) | 0 (0%) | 0 (0%) | 0 (0%) | 81 (20.3%) |
| Paramedical Staff | 11 (15.5%) | 22 (17.2%) | 0 (0%) | 0 (0%) | 0 (0%) | 0 (0%) | 0 (0%) | 33 (8.3%) |
| Support Staff | 16 (22.5%) | 33 (25.8%) | 0 (0%) | 0 (0%) | 0 (0%) | 0 (0%) | 0 (0%) | 49 (12.3%) |
| **Education** |  |  |  |  |  |  |  |  |
| 0 to primary | 0 (0%) | 0 (0%) | 0 (0%) | 11 (23.4%) | 21 (27.6%) | 14 (25.0%) | 1 (12.5%) | 47 (11.8%) |
| Up to class 7 | 7 (9.9%) | 8 (6.3%) | 1 (7.1%) | 10 (21.3%) | 10 (13.2%) | 7 (12.5%) | 1 (12.5%) | 44 (11.0%) |
| Up to class 10 | 22 (31.0%) | 43 (33.6%) | 3 (21.4%) | 4 (8.5%) | 15 (19.7%) | 10 (17.9%) | 0 (0%) | 97 (24.3%) |
| Up to class 12 | 0 (0%) | 0 (0%) | 1 (7.1%) | 10 (21.3%) | 17 (22.4%) | 11 (19.6%) | 2 (25.0%) | 41 (10.3%) |
| College degree/Diploma | 38 (53.5%) | 64 (50.0%) | 9 (64.3%) | 12 (25.5%) | 13 (17.1%) | 14 (25.0%) | 4 (50.0%) | 154 (38.5%) |
| Postgraduate | 4 (5.6%) | 13 (10.2%) | 0 (0%) | 0 (0%) | 0 (0%) | 0 (0%) | 0 (0%) | 17 (4.3%) |
| **Received COVID-19 vaccine after April 15** |  |  |  |  |  |  |  |  |
| No | 0 (0%) | 0 (0%) | 0 (0%) | 44 (93.6%) | 69 (90.8%) | 3 (5.4%) | 2 (25.0%) | 118 (29.5%) |
| Yes | 0 (0%) | 3 (2.3%) | 0 (0%) | 3 (6.4%) | 7 (9.2%) | 53 (94.6%) | 6 (75.0%) | 72 (18.0%) |
| Response not provided | 71 (100%) | 125 (97.7%) | 14 (100%) | 0 (0%) | 0 (0%) | 0 (0%) | 0 (0%) | 210 (52.5%) |
| **Currently have Children Aged 0-5 years** |  |  |  |  |  |  |  |  |
| No | 32 (45.1%) | 56 (43.8%) | 0 (0%) | 17 (36.2%) | 25 (32.9%) | 41 (73.2%) | 5 (62.5%) | 176 (44.0%) |
| Yes | 39 (54.9%) | 72 (56.3%) | 14 (100%) | 30 (63.8%) | 51 (67.1%) | 15 (26.8%) | 3 (37.5%) | 224 (56.0%) |
| **Children received essential immunization** |  |  |  |  |  |  |  |  |
| No | 11 (15.5%) | 20 (15.6%) | 6 (42.9%) | 5 (10.6%) | 15 (19.7%) | 1 (1.8%) | 3 (37.5%) | 61 (15.3%) |
| Yes | 32 (45.1%) | 56 (43.8%) | 0 (0%) | 17 (36.2%) | 25 (32.9%) | 41 (73.2%) | 5 (62.5%) | 176 (44.0%) |
| Not applicable | 28 (39.4%) | 52 (40.6%) | 8 (57.1%) | 25 (53.2%) | 36 (47.4%) | 14 (25.0%) | 0 (0%) | 163 (40.8%) |
| **Block of residence** |  |  |  |  |  |  |  |  |
| Not asked | 71 (100%) | 128 (100%) | 0 (0%) | 0 (0%) | 0 (0%) | 1 (1.8%) | 0 (0%) | 200 (50.0%) |
| Mawlai | 0 (0%) | 0 (0%) | 3 (21.4%) | 22 (46.8%) | 49 (64.5%) | 9 (16.1%) | 0 (0%) | 83 (20.8%) |
| Mawpat | 0 (0%) | 0 (0%) | 3 (21.4%) | 0 (0%) | 0 (0%) | 20 (35.7%) | 0 (0%) | 23 (5.8%) |
| Mylliem | 0 (0%) | 0 (0%) | 8 (57.1%) | 21 (44.7%) | 26 (34.2%) | 12 (21.4%) | 8 (100%) | 75 (18.8%) |
| Mawryngkneng | 0 (0%) | 0 (0%) | 0 (0%) | 4 (8.5%) | 1 (1.3%) | 14 (25.0%) | 0 (0%) | 19 (4.8%) |
