# Supplementary material for: Understanding COVID-19 vaccine hesitancy in Meghalaya, India: Multiple correspondence and agglomerative hierarchical cluster analyses
Source: PLOS Glob Public Health. 2024 Feb 27;4(2):e0002250. doi: 10.1371/journal.pgph.0002250 (PMC10898751; doi:10.1371/journal.pgph.0002250)
Supplement: Attachment — Submitted filename: Megh_Response to editor and reviewers_final.docx [file pgph.0002250.s004.docx]

EDITOR'S COMMENTS:

Journal Requirements:

1. Please review your reference list to ensure that it is complete and correct. If you have cited papers that have been retracted, please include the rationale for doing so in the manuscript text, or remove these references and replace them with relevant current references. Any changes to the reference list should be mentioned in the rebuttal letter that accompanies your revised manuscript. If you need to cite a retracted article, indicate the article’s retracted status in the References list and also include a citation and full reference for the retraction notice.

-> We confirm that our reference list is complete and correct.

2. In the ethics statement in the Methods, you have specified that verbal consent was obtained. Please provide additional details regarding how this consent was documented and witnessed, and state whether this was approved by the IRB.

🡪 Thank you for this comment. We have clarified the informed consent process and the IRB approval of the study in Lines 155-159 and Lines 173-177.

3. Please include a complete copy of PLOS’ questionnaire on inclusivity in global research in your revised manuscript. Our policy for research in this area aims to improve transparency in the reporting of research performed outside of researchers’ own country or community. The policy applies to researchers who have travelled to a different country to conduct research, research with Indigenous populations or their lands, and research on cultural artefacts. The questionnaire can also be requested at the journal’s discretion for any other submissions, even if these conditions are not met. Please find more information on the policy and a link to download a blank copy of the questionnaire here: https://journals.plos.org/globalpublichealth/s/best-practices-in-research-reporting. Please upload a completed version of your questionnaire as Supporting Information when you resubmit your manuscript.

🡪 A completed questionnaire has now been added to the Supporting Information (S4 Checklist) and cited in the manuscript (Lines 176-177).

4. Please send a completed 'Competing Interests' statement, including any COIs declared by your co-authors. If you have no competing interests to declare, please state "The authors have declared that no competing interests exist". Otherwise please declare all competing interests beginning with twhe statement "I have read the journal's policy and the authors of this manuscript have the following competing interests:"

🡪 The Competing Interests statement has been modified as follows:

We have read the journal's policy and the authors of this manuscript have the following competing interests:

1) Dr. Rajiv Sarkar serves as an Academic Editor to PLOS Global Public Health.
2) The rest of the authors declared no competing interests.

5. Please provide separate figure files in .tif or .eps format only and remove any figures embedded in your manuscript file. Please also ensure all files are under our size limit of 10MB.

🡪 We have removed all the figures embedded in the manuscript and provided them as separate files in .tif format.

For more information about figure files please see our guidelines:

https://journals.plos.org/globalpublichealth/s/figures

https://journals.plos.org/globalpublichealth/s/figures#loc-file-requirement

6. We have noticed that you have uploaded Supporting Information files, but you have not included a list of legends. Please add a full list of legends for your Supporting Information files after the references list.

🡪 A full list of legends has now been added after the reference list.

7. In the online submission form, you indicated that "The data that support the findings of this study are available on request from the corresponding author, SA". All PLOS journals now require all data underlying the findings described in their manuscript to be freely available to other researchers, either 1. In a public repository, 2. Within the manuscript itself, or 3. Uploaded as supplementary information.

This policy applies to all data except where public deposition would breach compliance with the protocol approved by your research ethics board. If your data cannot be made publicly available for ethical or legal reasons (e.g., public availability would compromise patient privacy), please explain your reasons by return email and your exemption request will be escalated to the editor for approval. Your exemption request will be handled independently and will not hold up the peer review process, but will need to be resolved should your manuscript be accepted for publication. One of the Editorial team will then be in touch if there are any issues.

🡪 We chose to provide the study data as supplementary information in Supporting information File 5.

Additional Editor Comments (if provided):

The manuscript has been reviewed and considered as an important contribution be included in the field to provide some understanding of the emerging challenges associated with Vaccine Hesitancy. However, to make the manuscript acceptable for publication, certain revisions have been suggested. Kindly pay attention to these suggestions as these would strengthen the manuscript more and clarify issues appropriately.

Title: The current title appears too long and needs modification. Suggested modification reads thus: "Elucidating the dynamics involved in COVID-19 Vaccine Hesitancy in Meghalaya, India: An Agglomerative Hierarchical Cluster Analysis".

🡪 Thank you for this suggestion. We have revised the title as follows: “Understanding COVID-19 Vaccine Hesitancy in Meghalaya, India: Multiple Correspondence Analysis and Agglomerative Hierarchical Cluster Analyses.”

Introduction: The last two sentences of the introduction mention "...The issue of vaccine hesitancy has become more apparent during a global pandemic and in the face of a new vaccine to address it" What science explains the dynamics involved in vaccine hesitancy? This has not featured in the study theoretical principles. It is expected that vaccine hesitancy is a personal-level disposition that triggers refusal or acceptance. The theoretical framework elucidating the disposition is completely omitted and not considered for evaluation in the study.

🡪 Thank you for your feedback regarding the theoretical framework used in our study on vaccine hesitancy. While the introduction briefly mentions the issue of vaccine hesitancy in the context of a global pandemic and a new vaccine, we agree that our study rationale can be further clarified to indicate the theoretically framework that guides the analysis. We have provided additional clarification in the Introduction section (Lines 119-127) and the Methods section (Lines 171-177) and hope that this addresses your comment.

Methods: The instrument development process appears missing including methods applied for assuring validity and reliability. The variables measured are not explicitly indicated in the methodology, but somewhere in figure 4 it gives the impression that negative perceptions of COVID-19 were measured. How was perception conceived and operationalized in the methodology of instrument development?

🡪 Thank you for your comment. We have provided a description of the COVID vaccine hesitancy questionnaire used in our study in the Methods (Lines 167 – 177) and included the questionnaire in the Supplementary material (Table S1). In the Discussion section, we indicated the limitations of the questionnaire employed in the study and the importance of using validated instruments to ensure the generalizability of findings (Lines 377-385).

[Note: HTML markup is below. Please do not edit.]

Reviewers' comments:

Reviewer #1: The manuscripts highlights important factors in the fight against COVID-19, particularly vaccine hesitancy and associated factors. This research is a thorough research that was done and the results well presented. The findings has potentials to impact public health policy and educational as well as health communication program design and implementation.

The objective of the research and manuscript is very clear. A robust statistical analysis procedures were used in analyzing and presenting the findings. Again, the authors have presented a discussion section that relates well with existing limited literature in the COVID-19 research and will had positive impact on knowledge and practices.

I do recommend that the language of the manuscript especially the methods section be examined again and effort needs to be made tp present the methods section with lay person's language and reading level. It appears to be verbose and use of technical terminology are used. This will make it a challenging for the general academic public to understand. Secondly, figures 2 and 4 don't appear clear and readers may not appreciate the importance of the messages in the figures. Please, make the figures more clear and simple to understand.

🡪 Thank you for this very valuable feedback. We appreciate your comments on the language and presentation of the manuscript. We believe that the use of technical terminology in the Methods section and the presentation of technical outputs in the Results section is essential to accurately convey the analytical framework and to facilitate the interpretation of the findings. However, to balance the technical detail in these sections with clarity and readability, we expanded the text in the Introduction and Discussion sections to provide the readers with more context for the interpretation of the results.

Overall, the author have presented a very elaborate research design and findings that only informs the Indian authorities and public about the determinants of covid-19 vaccine hesitancy, but the whole world, especially in developing countries where more misinformation, disinformation, and cultural barriers are reported to be challenges of health behavior.

🡪 Thank you for this encouraging comment.

Reviewer #2: Overall, the article is well-structured and informative. However, there are a few areas where improvements could be made for publication.

Title of the study

• It's a bit long and detailed, which might make it less reader-friendly. Consider simplifying it while retaining the essential information.

🡪 Thank you for this suggestion. We have modified the title as: “Understanding COVID-19 Vaccine Hesitancy in Meghalaya, India: A Multiple Correspondence Analysis and Agglomerative Hierarchical Cluster Analyses”.

Abstract

• Ensure that the abstract is concise while retaining clarity. Some sentences, such as those introducing the identified clusters, could be streamlined for brevity without sacrificing essential information.

🡪 Thank you for this suggestion. We have revised the text on the identified clusters for brevity.

• Specify the timeframe of the survey administration. Knowing when the survey was conducted is important, especially when dealing with evolving situations like the COVID-19 pandemic.

🡪 Thank you for this suggestion. We have added this crucial information (May 2021) to the abstract.

Background of the study

• The mention of disparities in the quality of COVID-19 data reporting is crucial, but the article could elaborate on how this may impact the understanding of vaccine hesitancy.

🡪 Thank you for this suggestion. We indicated that such disparities could have also impacted an adequate understanding of the extent of vaccine hesitancy in less prosperous states such as Meghalaya in the Introduction section (Lines 76-88).

• While the authors acknowledge regional disparities in COVID-19 infection and vaccination rates, they could delve deeper into the specific factors contributing to these disparities, beyond stating that they are highly clustered in more urbanized and prosperous states.

🡪 Thank you for this suggestion. We briefly summarized the factors contributing to these disparities in the Introduction section (Lines 79-82).

• While the study provides valuable insights into vaccine hesitancy, incorporating a theoretical framework would have enhanced the robustness and interpretability of the findings by offering a structured lens through which to analyze and understand the complex factors influencing vaccine hesitancy in diverse subpopulations.

🡪 Thank you for your feedback regarding the theoretical framework in our study on vaccine hesitancy. While the introduction briefly mentions the issue of vaccine hesitancy in the context of a global pandemic and a new vaccine, we agree that our study rationale can be further clarified to indicate the theoretically framework that guides the analysis. We have provided additional clarification in the Introduction section (Lines 119-127) and the Methods section (Lines 171-177) and hope that this addresses your comment.

Method

• The study period is mentioned as May 2021, but the recruitment of participants is stated as May 1st, 2022, to May 22th, 2022. There seems to be a discrepancy; please clarify the correct dates for the study period and recruitment.

🡪 Thank you for catching this typo. It is now fixed.

• It would be helpful to provide information on the sample size determination process and power analysis to ensure that the study has sufficient statistical power to detect meaningful differences within subgroups.

🡪 Thank you for your suggestion. The descriptive nature of the methods employed in our study did not necessitate statistical power calculations. However, we focused on ensuring a balanced and accurate representation of the target population, encompassing vaccine hesitant healthcare workers and community members. To achieve this, we employed a detailed stratified random sampling approach, as elaborated in the Methods section (Lines 146-155).

• While telephonic interviews were necessary due to pandemic-related restrictions, acknowledge and discuss potential limitations introduced by this mode of data collection.

🡪 Thank you for this great suggestion. We have acknowledged and discussed these limitations in the Discussion section (Lines 374-377).

• The dichotomous (yes or no) response format for the 19 questions might oversimplify the complexity of vaccine hesitancy.

🡪 Thank you for this great suggestion. We have acknowledged and discussed this limitation in the Discussion section (Lines 377-385).

• While verbal consent is mentioned, it would be beneficial to elaborate on the specifics of the consent process, ensuring participants fully understand the purpose of the study, their rights, and the potential implications of their participation.

🡪 Thank you for this comment. We have clarified the process of informed consent and the IRB approval in Lines 155-160.

• While you've described the methods used in data analysis (e.g., agglomerative hierarchical clustering based on Ward’s method), providing a brief rationale or justification for choosing these methods would add depth to the paper. This could help readers understand why these specific analytical approaches were employed.

🡪 Thank you for this suggestion. We added a sentence to provide justification for the chosen methods (Lines 185-189).

Results

• It might be helpful to use subheadings to distinguish between different sections of the results, making it easier for readers to follow the flow of information.

🡪 Thank you for this comment. Our results section is now divided into three subsections with the subheadings 1) Study sample and data collection, 2) Multiple correspondence analysis, and 3) Agglomerative hierarchical clustering.

• For each cluster, provide more context on why certain characteristics or attitudes are prevalent within that group. This could involve a deeper exploration of the socio-cultural context in Meghalaya that may contribute to these patterns of vaccine hesitancy.

🡪 Thank you for your suggestion. We have provided a more detailed interpretation of our findings in the Discussion section, while maintaining the Results section to present the findings directly from our analysis.

• Use consistent terminology throughout the paper. For example, ensure that terms like "undecisive" are used consistently, and consider alternatives such as "indecisive" for clarity.

🡪 This is now corrected (undecisive -> indecisive) across the manuscript.

Discussion of Findings and Addressing Study Limitations

• Explore further the gender and age dynamics within clusters, especially Cluster 2 with female healthcare workers and Cluster 5 with senior participants. Provide insights into how age and gender intersect with vaccine hesitancy.

🡪 Thank you. This point is now further elaborated in the Discussion section (Lines 346-359)

• Given that the study was conducted in May 2021, consider discussing the relevance and timeliness of the findings in the context of the evolving COVID-19 situation. This is particularly important if there have been significant changes in vaccine distribution, public perception, or policies since the study period.

🡪 Thank you for this great suggestion. We elaborated on this point in the last paragraph of the Discussion section (Lines 391 – 405).

• Acknowledge the study's limitations, such as the lack of data representative of the entire population in Meghalaya and the use of non-validated survey instruments. Discuss how these limitations might affect the generalizability of the findings.

🡪 Thank you for this suggestion. We have elaborated further on the limitations of our study as per your comment (Lines 367-396). We did not specifically highlight the lack of representativeness of the general population in Meghalaya as a limitation because our study explicitly defined the target population as ‘vaccine-eligible’ and ‘vaccine-hesitant’ individuals in Shillong city, Meghalaya, during May 2021, from two specific subgroups (i.e., healthcare workers and community members).

Proposing Future Research Directions:

• Suggest potential avenues for future research based on the gaps identified in this study.

🡪 Thank you for your suggestion. We have outlined several potential directions for future research in the Discussion section, building upon the identified limitations of the study (Lines 367-385)
